# Evaluating the Field 2-Point Method for the Relative Load-Velocity Relationship Monitoring in Free-Weight Back Squats

**DOI:** 10.5114/jhk/193975

**Published:** 2024-12-19

**Authors:** Zongwei Chen, Xiuli Zhang, Amador García-Ramos

**Affiliations:** 1 School of Physical Education and Sports Science, South China Normal University, Guangzhou, China.; 2 Department of Physical Education and Sport, Faculty of Sport Sciences, University of Granada, Granada, Spain.; 3 Department of Sports Sciences and Physical Conditioning, Faculty of Education, Universidad Católica de la Santísima Concepción, Concepción, Chile.

**Keywords:** exercise intensity, velocity-based training, field conditions

## Abstract

This study investigated the between-session variability and concurrent validity of the relative load-velocity relationship obtained from different methods during the free-weight back squat. In counterbalanced order, 39 resistance-trained male participants performed two sessions with six different loads (i.e., a multiple-point test) and two sessions with two different loads (i.e., a 2-point test) followed by the actual one-repetition maximum (1RM) attempts. The mean velocity (MV) corresponding to various %1RMs (at every 5% interval from 40 to 90%1RM) was determined through individualized linear regression models using three methods: (i) multiple-point: data of ~40, 50, 60, 70, 80, and 90%1RM from the multiple-point test, (ii) non-field 2-point: data of the lightest and heaviest loads from the multiple-point test, and (iii) field 2-point: data of ~40 and 90%1RM from the 2-point test. The main findings revealed that the between-session variability of the MVs derived from the %1RM-MV relationships was low (absolute differences = 0.02‒0.03 m•s^−1^) and similar (p = 0.074‒0.866) across the three methods. Additionally, when compared to the multiple-point method, both the non-field and field 2-point methods showed high correlations (pooled r across all %1RMs = 0.95 ± 0.01 and 0.72 ± 0.09, respectively) and small systematic biases (ranging from −0.01 to 0.01 m•s^−1^). Therefore, we recommend that strength and conditioning practitioners use the %1RM-MV relationship, modeled by the field 2-point method, as a quicker and fatigue-free procedure for prescribing the relative load during the free-weight back squat. Specifically, a light load near 40%1RM and a heavy load near 90%1RM are suggested for this method.

## Introduction

Resistance training serves as an important approach for enhancing strength, power, and sport performance (Suchomel et al., 2016). Training intensity is widely regarded by strength and conditioning scientists as one of the most crucial variables for achieving desirable physiological adaptations through resistance training. It is commonly identified by the percentage of the maximum load that can be lifted only once with full range of motion in a given exercise, also known as the percentage of the one-repetition maximum (%1RM) (Bird et al., 2005). The traditional method of prescribing the %1RM requires direct assessment of 1RM, typically involving a time-consuming, injury-prone, and highly fatiguing procedure consisting of progressively increasing loads until failure (Niewiadomski et al., 2008). Additionally, fluctuations in 1RM could occur as a result of training and non-training-related stressors such as nutrition, sleep or daily stress, potentially resulting in uncorrected short-term %1RM monitoring (Brotherton et al., 2019; Byrd et al., 2018). To address the limitations associated with direct 1RM testing, utilizing lifting velocity has emerged as a feasible method for estimating 1RM and determining the corresponding relative load-velocity (%1RM-V) relationship (García-Ramos, 2023b).

Earlier studies proposed the use of generalized %1RM-V relationship equations, often derived from polynomial regression models, to estimate the %1RM being lifted as soon as a repetition is performed with maximal concentric effort (Conceição et al., 2016; González-Badillo et al., 2010). The basic premise of generalized %1RM-V relationship equations is that the velocity corresponding to a specific %1RM for a given exercise should exhibit minimal between-subjects variability. However, recent studies have observed that the individualized %1RM-V relationship equations determined through linear regression models can provide more accurate %1RM estimation compared to polynomial regression models (Banyard et al., 2018; Pestaña-Melero et al., 2017). More recently, some researchers (García-Ramos et al., 2018a; Pérez-Castilla et al., 2023) proposed a simplified establishment of the individualized absolute load-velocity (L-V) relationship using only two loads (i.e., 2-point method) based on the high linearity of the L-V relationship to maximize efficiency and minimize fatigue during the testing procedure (García-Ramos, 2023a).

The 2-point method has been applied to establish the L-V relationship for estimating 1RM and assessing maximal neuromuscular capacities (García-Ramos, 2023a). For instance, García-Ramos et al. (2018a) found a trivial error between the actual 1RM and 1RM estimated by the 2-point method during the Smith machine bench press. Pérez-Castilla et al. (2023) reported that the 2-point method was a feasible approach for assessing the lower-limb maximal neuromuscular capacities. However, there is currently no research exploring the feasibility of establishing the %1RM-V relationship using the 2-point method. If the feasibility of the 2-point method is supported, it could potentially enhance the efficiency and reduce the level of fatigue during establishing the %1RM-V relationship. Furthermore, almost all previous studies employ the multiple-point incremental load testing procedure (i.e., testing more than two loads) followed by the analysis of data from only two loads (i.e., applied under non-field conditions) to establish the L-V relationship, which deviates from resistance training practice. Therefore, it would be more informative to test whether the 2-point method is feasible for establishing the %1RM-V relationship when applied under field conditions (i.e., collecting data at just two loads).

The multiple-point method has the capacity to establish the %1RM-V relationship due to its high between-session reliability and close-to-perfect goodness of fit (R^2^) (García-Ramos et al., 2019; Pestaña-Melero et al., 2017). Therefore, the prerequisite for the application of the field 2-point method is that the between-session variability and concurrent validity of the %1RM-V relationship modeled by such a method are comparable with respect to the multiple-point method. Miras-Moreno et al. (2023b) recently found that the 2-point testing procedure could lead to less cumulative fatigue, which means that an individual may achieve a higher 1RM following the 2-point testing procedure compared to the multiple-point testing procedure. The %1RM associated with the same absolute load in the 2-point procedure may be lower than that in the multiple-point procedure, but the movement velocities associated with the lightest absolute testing load should be the same due to consistent warm-up sets. As a result, the %1RM-V relationship derived from the multiple-point and field 2-point methods may differ. Consequently, it is unclear whether the %1RM-V relationship derived from the field 2-point method can be utilized as a quicker and fatigue-free alternative compared to the multiple-point method for relative load prescription.

Therefore, the main purpose of this study was to (I) compare the between-session variability of the mean velocity (MV) corresponding to a series of %1RMs between the multiple-point, non-field 2-point, and field 2-point methods, and (II) examine the concurrent validity of the non-field and field 2-point methods with respect to the multiple-point method for determining the MV corresponding to various %1RMs. We hypothesized that (I) the between-session variability for the MV across various %1RMs would not be significantly different between the different methods, based on the consistent reliability previously reported in studies for variables derived from the L-V relationship, comparing both the non-field and field 2-point methods with the multiple-point method (García- Ramos et al., 2018c; García-Ramos, 2023a). However, (II) we anticipated that the concurrent validity would be higher for the non-field 2-point method (e.g., lower systematic and random errors identified by Bland-Altman analysis), as it utilizes the same two extreme experimental points and 1RM that are also employed in the multiple-point method.

## Methods

### Participants

Thirty-nine resistance-trained male participants (age: 21.2 ± 1.7 years, body mass: 74.0 ± 8.7 kg, body height: 175.7 ± 3.9 cm, resistance training experience: 3.8 ± 1.9 years, preliminary free-weight back squat 1RM: 138.9 ± 20.8 kg, preliminary 1RM/body mass ratio: 1.88 ± 0.19) volunteered for this study. Prior to data collection sessions, they were instructed to participate in three familiarization sessions in which they performed the free-weight back squat at maximal intended concentric velocity against light (i.e., MV > 1.00 m•s^−1^), moderate (i.e., 0.60 m•s^−1^ ≤ MV ≤ 1.00 m•s^−1^), and heavy loads (MV < 0.60 m•s^−1^) (Sánchez-Medina et al., 2017). Each session was separated by 72 h and consisted of two sets of light loads (5 repetitions/set), two sets of moderate loads (3 repetitions/set), and two sets of heavy loads (1 repetition/set). The first data collection session commenced 72 h after the last familiarization session. Participants reported no physical limitations or musculoskeletal injuries that could compromise their squat performance. They were instructed to abstain from vigorous exercise the day before each testing session. Additionally, they were briefed on the study procedures and provided written informed consent prior to participation. The study protocol complied with the principles of the Declaration of Helsinki and received approval from the Institutional Review Board of the South China Normal University (protocol code: SCNU-SPT-2023-171; approval date: 01 December 2023).

### Design and Procedures

A counterbalanced repeated-measures design was used to compare the between-session variability of the three methods for establishing the %1RM-V relationship during the free-weight back squat, and to investigate the concurrent validity of the non-field and field 2-point methods with respect to the multiple-point method for determining the MV corresponding to various %1RMs. After an initial 1RM test (T1), participants performed four experimental sessions (T2‒T5) over two consecutive weeks. Using a random sequence generated by Excel 2019 software (Microsoft, Redmond, USA), half of the participants undertook the sessions in the order of the multiple-point test (i.e., 40, 50, 60, 70, 80, and 90% of 1RM from T1 were applied), the 2-point test (i.e., 40 and 90% of 1RM from T1 were applied), the 2-point test, and the multiple-point test, while the other half undertook the sessions in the opposite order (i.e., the 2-point test, the multiple-point test, the multiple-point test, and the 2-point test). The counterbalanced design was chosen to ensure that any observed differences were not due to improved performance or reliability from training effects or increased familiarity with the testing procedures in later sessions. The 1RM was also directly measured in T2‒T5 to construct the %1RM-V relationships. Each testing session was separated by 72 h of rest and conducted at the same time of the day for each participant (± 1 h) under similar environmental conditions (~21°C and ~60% humidity) (Haischer et al., 2023).

### Measures

#### Preliminary 1RM Test (T1)

Participants started with a standardized warm-up protocol, including 3 min of jogging at a pace of 6 km•h^−1^ on a treadmill (ShuHua Sports, Quanzhou, China), a series of dynamic stretching exercises, and 10 repetitions of squatting using an empty barbell. After a 3-min rest interval, they performed an incremental load testing procedure comprising five attempts at 50% of their self-reported 1RM, three attempts at 70% of their self-reported 1RM, and one attempt at 90% of their self-reported 1RM. This was followed by attempts at progressively heavier loads ranging from 5.0 to 0.5 kg until they achieved their actual 1RM (Haff et al., 2019; Suchomel et al., 2024). Rest intervals of approximately 3‒5 min were provided between sets, and participants were given the opportunity to retry if they failed.

#### Experimental Testing Sessions (T2‒T5)

All experimental testing sessions began with a standardized warm-up protocol consistent with T1, including 3 min of jogging at a pace of 6 km•h^−1^ on a treadmill, a series of dynamic stretching exercises, and 10 repetitions of squatting using an empty barbell. After a 3-min rest interval, participants performed an incremental load testing procedure with six different loads (40, 50, 60, 70, 80, and 90% of 1RM from T1) or only two different loads (40 and 90% of 1RM from T1) followed by the actual 1RM attempts following the procedure described for T1 (i.e., attempting progressively heavier loads ranging from 5.0 to 0.5 kg until reaching the actual 1RM). The selection of testing loads was based on a criterion with the lightest load being moderate and the heaviest load approaching 1RM (García-Ramos, 2023a). The rest interval between loads was set to 3‒5 min (3 min for approximately 40‒90%1RM and 5 min for heavier loads), and the repetitions of each load were set to 3 (~40, 50, and 60%1RM), 2 (~70 and 80%1RM), or 1 (~90%1RM and heavier loads). A 5-s inter-repetition rest interval was provided for loads with more than 1 repetition. Only the repetition with the fastest MV of each load was collected for data analyses.

#### Description of the Tested Exercise

The tested exercise (i.e., the free-weight back squat) was performed with a high bar configuration and technique which required participants initially to stand upright with their hips and knees locked, feet approximately shoulder-width apart, and the barbell rested across their upper back. Upon the command of “squat”, they descended with a self-selected controlled speed (~1.2 s) until the middle of their thighs (i.e., the thighbone) reached the position parallel to the ground. Subsequently, utilizing the stretch-shortening cycle, they were encouraged to return immediately to the starting position with maximal intended velocity and received auditory MV feedback immediately after completing each attempt (Weakley et al., 2018, 2023). A series of non-elastic cardboard markers was placed under the participants’ buttocks to define the individualized range of motion (i.e., the lowest point of the squat was defined as the parallel position between the thighbone and the ground for each participant), which was recorded during the first familiarization session and maintained consistent throughout all data collection sessions (T1‒T5). Participants were instructed to keep the barbell in constant contact with their upper back, avoid leaving the ground at the end of the movement, and refrain from wearing weight belts during any repetition to prevent fluctuations in MV (Fong et al., 2022).

#### Measurement Equipment and Data Analysis

At the start of the preliminary 1RM test, body mass was measured using an InBody 270 device (Biospace, California, USA) and body height was determined with a stadiometer (Yunpeng Technology, Dalian, China). An Olympic barbell (20 kg; ShuHua Sports, Quanzhou, China) and a squat rack (CrossMax Sports, Weifang, China) were used in all testing sessions coupled with a valid linear position transducer (GymAware PowerTool, Kinetic Performance Technology, Canberra, Australia) (Weakley et al., 2021a). This transducer was positioned on the ground to the right of the participants’ feet, with the Velcro strap attached 50 cm to the right of the barbell center. The MV (i.e., the mean velocity from the beginning of the concentric phase until the barbell reaches its maximum height (García-Ramos et al., 2018b)) was assessed with the GymAware software version 4.1.2 on a Ninth Generation Apple iPad (Apple Inc., California, USA).

The %1RM-V relationships were modeled using three different methods with individual linear regression: multiple-point (i.e., data of six loads from the multiple-point test), non-field 2-point (i.e., data of the lightest and heaviest loads from the multiple-point test), and field 2-point (i.e., data of two loads from the 2-point test). The absolute loads lifted in T2‒T5 were expressed as a percentage of the 1RM directly measured in the same session. Following this, a linear regression model was used to determine the individualized %1RM-V relationship, formulated as MV = (slope × %1RM) + velocity intercept. This calculation was based on the fastest MV observed for each load lifted. Subsequently, the MVs corresponding to a series of %1RMs, specifically at every 5% interval from 40 to 90%1RM, were calculated.

### Statistical Analysis

Descriptive data, presented as means and standard deviation, were assessed for normal distribution using the Shapiro-Wilk test (*p* > 0.05). Paired samples *t* tests and Cohen’s *d* effect size (ES) were used to compare the collected data (actual 1RM, actual %1RM represented by the submaximal loads lifted, and MV attained for the submaximal loads) between the multiple-point and 2-point tests separately for the first (T2 vs. T3) and second (T4 vs. T5) testing sessions of each test. The strength of the %1RM-V relationship was assessed through the R^2^. A 2-way repeated-measures analysis of variance (ANOVA) with the factors of “load” (40 vs. 45 vs. 50 vs. 55 vs. 60 vs. 65 vs. 70 vs. 75 vs. 80 vs. 85 vs. 90% of 1RM) and “method” (multiple-point vs. non-field 2-point vs. field 2-point). Bonferroni post hoc corrections were applied to the absolute differences of the MVs corresponding to each %1RM between the first and second sessions. The concurrent validity of the non-field and field 2-point methods (considering the average values calculated from the first and second sessions) with respect to the multiple-point method was examined through paired samples *t* tests, ES, the Pearson’s correlation coefficient (*r*), and Bland-Altman 95% limits of agreement (i.e., 95%LoA; bias ± 1.96 × SD). The partial eta squared (*η_p_^2^*) was calculated for the ANOVA and interpreted as: trivial (< 0.010), small (0.010‒0.059), moderate (0.060‒0.140), and large (> 0.140) (Cohen, 1998). The ES was categorized as: trivial (< 0.20), small (0.20‒0.59), moderate (0.60‒1.19), large (1.20‒2.00), and very large (> 2.00). The *r* was interpreted as: trivial (< 0.10), small (0.10‒0.29), moderate (0.30‒0.49), large (0.50‒0.69), very large (0.70‒0.89), nearly perfect (0.90‒0.99), and perfect (1.00) (Hopkins et al., 2009). Statistical analyses were conducted using SPSS 25.0 software (SPSS Ins., Chicago, USA), with the alpha level set at 0.05.

## Results

The characteristics of the four experimental testing sessions are presented in Table 1. The main consistent difference across both comparisons (T2 vs. T3 and T4 vs. T5) was the greater MV recorded at ~90%1RM for the 2-point test compared to the multiple-point test (*p* ≤ 0.012, ES = 0.43‒0.63), while the MV corresponding to ~40%1RM was always comparable for both testing procedures (*p*=0.295‒.437, ES ranging from −0.17 to 0.13).

**Table 1:** Characteristics of the four experimental testing sessions.

Testing session	Load 1	Load 2	Load 3	Load 4	Load 5	Load 6	1RM
***First multiple-point test***							
Load, kg	55.6 ± 8.3	69.5 ± 10.4	83.4 ± 12.5	97.2 ± 14.6	111.2 ± 16.6	125.0 ± 18.7	139.3 ± 20.5
Load, %1RM	39.9 ± 1.6	49.9 ± 2.0	59.9 ± 2.4	69.8 ± 2.8	79.8 ± 3.2	89.8 ± 3.6	100
Velocity, m•s^−1^	0.97 ± 0.05	0.88 ± 0.05	0.79 ± 0.05	0.69 ± 0.05	0.59 ± 0.04	0.48 ± 0.05	0.28 ± 0.05
***First 2-point test***							
Load, kg	55.6 ± 8.3	125.0 ± 18.7					**141.1 ± 20.6**
Load, %1RM	**39.2 ± 1.5**	**88.3 ± 3.2**					100
Velocity, m•s^−1^	0.96 ± 0.04	**0.51 ± 0.04**					0.29 ± 0.06
***Second multiple-point test***							
Load, kg	55.6 ± 8.3	69.5 ± 10.4	83.4 ± 12.5	97.2 ± 14.6	111.2 ± 16.6	125.0 ± 18.7	142.0 ± 21.3
Load, %1RM	39.1 ± 1.6	49.0 ± 2.0	58.8 ± 2.4	68.5 ± 2.8	78.4 ± 3.2	88.1 ± 3.6	100
Velocity, m•s^−1^	0.98 ± 0.05	0.89 ± 0.05	0.80 ± 0.05	0.71 ± 0.05	0.61 ± 0.05	0.50 ± 0.05	0.28 ± 0.05
***Second 2-point test***							
Load, kg	55.6 ± 8.3	125.0 ± 18.7					143.1 ± 21.1
Load, %1RM	38.8 ± 1.5	87.4 ± 3.4					100
Velocity, m•s^−1^	0.99 ± 0.05	**0.52 ± 0.05**					0.28 ± 0.05

Data are presented as means ± standard deviation; 1RM indicates one-repetition maximum;

Bold values: significant differences compared to the multiple-point test (p < 0.05)

**Figure 1:**
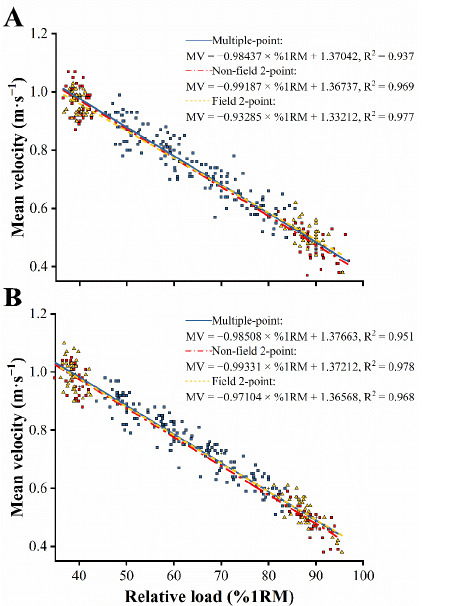
Generalized across the participants’ relationship between the relative load (%1RM) and mean velocity (MV) during the first (A) and second (B) sessions. The multiple-point (blue solid line), non-field 2-point (red dash-dotted line), and field 2-point (yellow dashed line) regression equations are depicted. R^2^ indicates goodness of fit.

The generalized %1RM-V relationships were nearly perfect and linear for all methods (Figure 1; R^2^ ≥ 0.937). The individualized %1RM-V relationships modeled by the multiple-point method also revealed a very high goodness of fit in the first (R^2^ = 0.949‒1.000) and second (R^2^ = 0.957‒0.990) testing sessions.

A very low between-session variability for the MV corresponding to each %1RM was observed for the three methods (absolute differences ≤ 0.03 m•s^−1^) (Table 2). ANOVA did not reveal any significant main effect or load × method interaction (F = 0.1‒3.1, *p* = 0.074‒0.866, *η_p_^2^* = 0.003‒0.076), suggesting that the variability was comparable for the three methods and for the range of loads examined (40 to 90% of 1RM).

A nearly perfect correlation for the whole %1RM-V relationship was observed between the non-field 2-point and multiple-point methods (*r* = 0.95 ± 0.01), while the systematic bias was consistently low (0.01 m•s^−1^). Similarly, a very large correlation for the whole %1RM-V relationship was observed between the field 2-point and multiple-point methods (*r* = 0.72 ± 0.09), while the systematic bias was consistently low (ranging from −0.01 to 0.01 m•s^−1^). However, the correlations between the multiple-point and field 2-point methods were greater for light-moderate loads (40‒70%1RM; *r* ≥ 0.75) compared to heavier loads (75‒90%1RM; *r* ≤ 0.71) (Table 3).

**Table 2:** Two-way repeated-measures analysis of variance (ANOVA) comparing the between-session variability (considering the absolute difference) of the relative load-velocity relationships between the loads and methods.

Load (%1RM)	Method			ANOVA
	Multiple-point (m•s^−1^)	Non-field 2-point (m•s^−1^)	Field 2-point (m•s^−1^)	
40	0.023 ± 0.018	0.029 ± 0.017	0.030 ± 0.030	Load: F = 3.1, *p* = 0.074, *η_p_^2^* = 0.076 Method: F = 2.2, *p* = 0.136, *η_p_^2^* = 0.055 Load × Method: F = 0.1, *p* = 0.866, *η_p_^2^* = 0.003
45	0.021 ± 0.016	0.027 ± 0.016	0.027 ± 0.028	
50	0.020 ± 0.015	0.024 ± 0.016	0.026 ± 0.026	
55	0.018 ± 0.014	0.023 ± 0.016	0.025 ± 0.024	
60	0.018 ± 0.013	0.022 ± 0.016	0.025 ± 0.022	
65	0.018 ± 0.013	0.022 ± 0.016	0.026 ± 0.021	
70	0.018 ± 0.013	0.023 ± 0.016	0.027 ± 0.021	
75	0.019 ± 0.015	0.024 ± 0.017	0.028 ± 0.022	
80	0.020 ± 0.017	0.026 ± 0.019	0.029 ± 0.024	
85	0.022 ± 0.019	0.028 ± 0.021	0.031 ± 0.027	
90	0.025 ± 0.020	0.030 ± 0.024	0.033 ± 0.030	

Data are presented as means ± standard deviation. %1RM indicates the percentage of the one-repetition maximum; F, F statistic; p, p-value obtained from two-way repeated-measures ANOVA; η_p_^2^, partial eta squared

**Table 3:** Concurrent validity of the non-field and field 2-point methods with respect to the multiple-point method in establishing the relative load-velocity relationship.

Method	Load (%1RM)	Multiple-point (m•s^−1^)	Two-point (m•s^−1^)	*p*	ES	*r* (95%CI)	Bias (95%LoA)
Non-field 2-point	40	0.98 ± 0.04	0.97 ± 0.04	**0.006**	0.47	0.96 (0.93, 0.98)	0.01 (−0.02, 0.03)
	45	0.93 ± 0.04	0.92 ± 0.04	**0.001**	**0.60**	0.96 (0.92, 0.98)	0.01 (−0.02, 0.03)
	50	0.88 ± 0.04	0.87 ± 0.04	**0.001**	**0.61**	0.96 (0.92, 0.98)	0.01 (−0.01, 0.03)
	55	0.83 ± 0.04	0.82 ± 0.03	**< 0.001**	**0.71**	0.96 (0.92, 0.98)	0.01 (−0.01, 0.03)
	60	0.78 ± 0.03	0.77 ± 0.03	**< 0.001**	**0.67**	0.95 (0.91, 0.98)	0.01 (−0.01, 0.03)
	65	0.73 ± 0.03	0.73 ± 0.03	**< 0.001**	**0.67**	0.95 (0.91, 0.97)	0.01 (−0.01, 0.03)
	70	0.68 ± 0.03	0.67 ± 0.03	**< 0.001**	**0.85**	0.95 (0.90, 0.97)	0.01 (−0.01, 0.03)
	75	0.63 ± 0.03	0.63 ± 0.03	**< 0.001**	**0.86**	0.94 (0.89, 0.97)	0.01 (−0.01, 0.03)
	80	0.59 ± 0.03	0.58 ± 0.03	**< 0.001**	**0.87**	0.93 (0.88, 0.96)	0.01 (−0.01, 0.03)
	85	0.54 ± 0.03	0.53 ± 0.03	**< 0.001**	**0.85**	0.93 (0.87, 0.96)	0.01 (−0.01, 0.03)
	90	0.49 ± 0.03	0.48 ± 0.03	**< 0.001**	**0.85**	0.92 (0.86, 0.96)	0.01 (−0.01, 0.03)
Field 2-point	40	0.98 ± 0.04	0.97 ± 0.04	**0.021**	0.39	0.76 (0.59, 0.87)	0.01 (−0.04, 0.07)
	45	0.93 ± 0.04	0.92 ± 0.04	**0.031**	0.36	0.78 (0.61, 0.88)	0.01 (−0.04, 0.06)
	50	0.88 ± 0.04	0.87 ± 0.03	**0.049**	0.33	0.79 (0.63, 0.88)	0.01 (−0.04, 0.05)
	55	0.83 ± 0.04	0.83 ± 0.03	0.082	0.29	0.80 (0.64, 0.89)	0.01 (−0.04, 0.05)
	60	0.78 ± 0.03	0.78 ± 0.03	0.276	0.18	0.79 (0.64, 0.89)	0.00 (−0.04, 0.05)
	65	0.73 ± 0.03	0.73 ± 0.03	0.766	0.05	0.78 (0.62, 0.88)	0.00 (−0.04, 0.04)
	70	0.68 ± 0.03	0.68 ± 0.03	0.820	0.04	0.75 (0.57, 0.86)	0.00 (−0.04, 0.04)
	75	0.63 ± 0.03	0.64 ± 0.03	0.941	−0.01	0.71 (0.51, 0.84)	0.00 (−0.04, 0.04)
	80	0.59 ± 0.03	0.59 ± 0.03	0.696	−0.06	**0.66 (0.43, 0.80)**	0.00 (−0.05, 0.04)
	85	0.54 ± 0.03	0.54 ± 0.03	0.474	−0.12	**0.59 (0.34, 0.77)**	0.00 (−0.05, 0.05)
	90	0.49 ± 0.03	0.49 ± 0.03	0.287	−0.17	**0.53 (0.26, 0.73)**	−0.01 (−0.06, 0.05)

Data are average values calculated from the first and second testing sessions and presented as means ± standard deviation. 1RM indicates one-repetition maximum; 95%CI, 95% confidence intervals; 95%LoA, 95% limits of agreement (bias ± 1.96 standard deviation); ES, Cohen’s d effect size ([Multiple-point ‒ 2-point] / standard deviation of the differences); p, p values obtained from paired sample t tests; r, Pearson’s correlation coefficient. Bold p-values indicate p < 0.05; bold ES-values, ES > 0.6; bold r-values, r < 0.7

## Discussion

This study was designed to compare the between-session variability of three methods (multiple-point, non-field 2-point, and field 2-point) for establishing the %1RM-V relationship during the free-weight back squat, and to investigate the concurrent validity of the non-field and field 2-point methods with respect to the multiple-point method for determining the MV corresponding to 40‒90% of 1RM. The main results of the present study revealed that: (I) the between-session variability of the MVs obtained from the %1RM-V relationship was low and similar across loads and methods, (II) both the non-field and field 2-point methods showed small systematic biases compared to the multiple-point method, while, as expected, the correlations were grater for the non-field 2-point method for it considered the same experimental point as the multiple-point method, and (III) the 2-point test demonstrated less levels of fatigue compared to the multiple-point test as revealed by the greater MV for the ~90%1RM in both sessions and greater 1RM in the first session. Therefore, this study supports the application of the field 2-point method for establishing the %1RM-V relationship as a feasible and fatigue-free alternative to the multiple-point method during the free-weight back squat.

It is extremely important that the %1RM-V relationship used for monitoring %1RM is obtained with an acceptable between-session reliability (Atkinson et al., 1998). Previous research has found a very high between-session reliability for the %1RM-V relationship established by the individual linear multiple-point method in exercises such as the prone bench pull (García-Ramos et al., 2019), the bench press (Pestaña-Melero et al., 2017), and the back squat (Banyard et al., 2018), implying that the multiple-point method could be used to accurately prescribe the relative loads from lifting velocity recordings (Weir, 2005). However, there are no previous studies comparing the between-session variability of the %1RM-V relationship among the multiple-point, non-field 2-point, and field 2-point methods. The results of the present study prospectively revealed that regardless of the %1RM-V relationship modeling method employed, the between-session variability in MVs was similar for each %1RM as shown by ANOVA which failed to detected any significant main effect (*p* = 0.074‒0.136) or load × method interaction (*p* = 0.866). The between-session variability was similar for all methods and lower than 0.06 m•s^−1^, which is the smallest detectable difference of MV reported by Banyard et al. (2018), Orange et al. (2020), and Rebelo et al. (2023) during the back squat exercise. This suggests that the non-field and field 2-point methods could serve as alternatives to the multiple-point method for establishing the %1RM-V relationship.

Another premise of modeling the %1RM-V relationship with the 2-point method could be to have great concurrent validity with respect to the multiple-point method, as the %1RM-V relationship modeled by the multiple-point method has been found to present nearly perfect R^2^ in previous research (Banyard et al., 2018; Thompson et al., 2021). In line with previous studies, we found R^2^ of the individualized %1RM-V relationship established by the multiple-point method ranging from 0.949 to 1.000. To our knowledge, the present study was the first to investigate the concurrent validity of the non-field and field 2-point methods in modeling the %1RM-V relationship compared to the multiple-point method. The main results of the present study indicate that the MVs corresponding to each %1RM were 0.01 m•s^−1^ lower for the non-field 2-point method compared to the multiple-point method with 95%LoA ranging from −0.02 to 0.03 m•s^−1^ and *r* coefficients ranging from 0.92 to 0.96. These findings further corroborate the minimal impact that intermediate points have on the final outcomes of the L-V and %1RM-V relationships when used in modeling (García-Ramos, 2023a). More importantly, the field 2-point method provided an overall very large correlation (*r* = 0.72 ± 0.09) and very low systematic bias (≤ 0.01 m•s^−1^) with respect to the multiple-point method. In this regard, it is important to consider that according to Orange et al. (2020) and Rebelo et al. (2023), a MV difference lower than 0.06 m•s^−1^ in the free-weight back squat implies a load difference lower than 5% of 1RM, which is negligible for long-term training-induced adaptation (Weakley et al., 2021b). Therefore, the non-field and field 2-point methods demonstrated acceptable concurrent validity for establishing the %1RM-V relationship in resistance training practices compared to the multiple-point method.

An intriguing result was that the correlations between the multiple-point and field 2-point methods were greater for light-moderate loads (40‒70%1RM; *r* ≥ 0.75) compared to heavier loads (75‒90%1RM; *r* ≤ 0.71). We found that the MVs at ~40%1RM (i.e., the first testing load) were consistently similar between the multiple-point and 2-point tests (*p* = 0.295‒0.437), which is not surprising because the same warm-up sets were employed. However, the %1RM associated with the first testing load tended to be lower in the 2-point test compared to the multiple-point test due to participants tended to achieve a higher 1RM during the 2-point test (especially in the first session). However, the MVs at ~90%1RM in the 2-point test were consistently higher than those in the multiple-point test due to less accumulated fatigue (*p* ≤ 0.012), which aligns with the findings of Miras-Moreno et al. (2023a) who found that lifting more than two loads could compromise the maximal neuromuscular capacities during the prone bench pull. These factors led to MVs obtained from the field 2-point method tending to be lower than the multiple-point method at light loads and higher than the multiple-point method at heavy loads, but there was a very large overall correlation (*r* = 0.72 ± 0.09) between these two methods. These factors also contributed to lower MVs obtained by the non-field 2-point method compared to the multiple-point method (*p* ≤ 0.006, ES ≥ 0.47). However, based on the existing evidence, a bias of 0.01 m•s^−1^ in MV was unlikely to impact the long-term physiological adaptations during back squat training (Banyard et al., 2018). We could still consider the non-field and field 2-point methods to have comparable concurrent validity with respect to the multiple-point method. Furthermore, it is crucial to highlight that the %1RM-V relationship modeled by the field 2-point method could be more consistent with resistance training practices, as typical resistance training sessions do not commence with a prolonged warm-up protocol consisting of six incremental loads lifted at maximal intended concentric velocity (Iversen et al., 2021; Tsoukos et al., 2023).

When interpreting the findings of this study, the following limitations should be considered. First, the findings of the present study may not be applicable to other exercises, given that prior research has shown that the stability of movement velocity is exercise-specific (McBurnie et al., 2019; Grgic et al., 2020). Free-weight lower-limb exercises engage more joints and muscles and demand a more intricate technique than upper-limb exercises. Specifically, when an individual has poor lifting technique, the velocity measurement device (e.g., GymAware PowerTool applied in this study) is more likely to overestimate the velocity in lower-limb exercises due to the barbell’s asymmetrical anterior-posterior and medial-lateral horizontal movement (McBurnie et al., 2019). Therefore, we anticipate that the field 2-point method could also be a feasible approach to establish the %1RM-V relationship during upper-limb exercises. Second, we did not examine the long-term variability of the %1RM-V relationship established by the non-field and field 2-point methods. This insight is practically valuable for coaches in determining how frequently the individualized %1RM-V relationship needs to be updated. Lastly, the following potential limitations may impact the generalizability of our findings regarding the implementation of the field 2-point method: the recruitment of a homogeneous sample of resistance-trained male participants and the highly standardized testing conditions. Therefore, future research should apply the field 2-point method in other populations and under other testing conditions to investigate its generalizability.

## Conclusions

The between-session variability of the MVs obtained from the %1RM-V relationship was low and comparable for the multiple-point, non-field 2-point, and field 2-point methods. Additionally, high correlations and small systematic biases existed between the (non-field and field) 2-point and multiple-point methods. Furthermore, the 2-point test demonstrated lower levels of fatigue compared to the multiple-point test. Therefore, the field 2-point method could be a feasible and fatigue-free alternative to the multiple-point method for establishing the %1RM-V relationship during the free-weight back squat. It is important to acknowledge, however, that our investigation was limited to young male resistance training participants and the free-weight back squat. Consequently, we cannot guarantee the generalizability of the field 2-point method under other testing conditions.

Based on the main findings of this study, the following steps are recommended for applying the field 2-point method: (I) performing a standardized warm-up before the formal testing procedure, including jogging, dynamic stretching exercises, and barbell squatting against a range of loads, (II) lifting only two loads and recording the corresponding MVs, one facilitating a MV close to 1.00 m•s^−1^ (light load at ~40%1RM) and another close to 0.50 m•s^−1^ (heavy load at ~90%1RM), (III) modeling an individual linear %1RM-V relationship, whether based on the actual or estimated 1RMs, to prescribe the training intensity during the free-weight back squat.

## References

[ref1] Atkinson, G. & Nevill, A. M. (1998). Statistical methods for assessing measurement error (reliability) in variables relevant to sports medicine. Sports Medicine, 26(4), 217–238. 10.2165/00007256-199826040-00002 9820922

[ref2] Banyard, H. G., Nosaka, K., Vernon, A. D. & Haff, G. G. (2018). The reliability of individualized load-velocity profiles. International Journal of Sports Physiology and Performance, 13(6), 763–769. 10.1123/ijspp.2017-0610 29140148

[ref3] Bird, S. P., Tarpenning, K. M. & Marino, F. E. (2005). Designing resistance training programmes to enhance muscular fitness-A review of the acute programme variables. Sports Medicine, 35(10), 841–851. 10.2165/00007256-200535100-00002 16180944

[ref4] Brotherton, E. J., Moseley, S. E., Langan-Evans, C., Pullinger, S. A., Robertson, C. M., Burniston, J. G. & Edwards, B. J. (2019). Effects of two nights partial sleep deprivation on an evening submaximal weightlifting performance; are 1 h powernaps useful on the day of competition? Chronobiology International, 36(3), 407–426. 10.1080/07420528.2018.1552702 30626222

[ref5] Byrd, M. T. & Bergstrom, H. C. (2018). Effects of very short-term dynamic constant external resistance exercise on strength and barbell velocity in untrained individuals. International Journal of Exercise Science, 11(1), 867–874. 29997728 10.70252/OZPL9042 PMC6033493

[ref6] Cohen, J. (1998). *Statistical Power Analysis for the Behavioral Sciences* (2nd ed.). Erlbaum.

[ref7] Conceição, F., Fernandes, J., Lewis, M., Gonzaléz-Badillo, J. J. & Jimenéz-Reyes, P. (2016). Movement velocity as a measure of exercise intensity in three lower limb exercises. Journal of Sports Sciences, 34(12), 1099–1106. 10.1080/02640414.2015.1090010 26395837

[ref8] Fong, S. S. M., Chung, L. M. Y., Gao, Y., Lee, J. C. W., Chang, T. C. & Ma, A. W. W. (2022). The influence of weightlifting belts and wrist straps on deadlift kinematics, time to complete a deadlift and rating of perceived exertion in male recreational weightlifters: An observational study. *Medicine*, 101(7), e28918. 10.1097/MD.0000000000028918 35363215 PMC9282110

[ref9] García-Ramos, A., Haff, G. G., Pestaña-Melero, F. L., Pérez-Castilla, A., Rojas, F. J., Balsalobre-Fernández, C. & Jaric, S. (2018a). Feasibility of the 2-point method for determining the 1-repetition maximum in the bench press exercise. *International Journal of Sports Physiology and Performance*, 13(4), 474–481. 10.1123/ijspp.2017-0374 28872384

[ref10] García-Ramos, A., Pestaña-Melero, F. L., Pérez-Castilla, A., Rojas, F. J. & Haff, G. G. (2018b). Mean velocity vs. mean propulsive velocity vs. peak velocity: Which variable determines bench press relative load with higher reliability? *Journal of Strength and Conditioning Research*, 32(5), 1273–1279. 10.1519/jsc.0000000000001998 28557855

[ref11] García-Ramos, A. & Jaric, S. (2018c). Two-point method: A quick and fatigue-free procedure for assessment of muscle mechanical capacities and the 1 repetition maximum. Strength and Conditioning Journal, 40(2), 54–66. 10.1519/SSC.0000000000000359

[ref12] García-Ramos, A., Ulloa-Díaz, D., Barboza-González, P., Rodríguez-Perea, Á., Martínez-García, D., Quidel-Catrilelbún, M., Guede-Rojas, F., Cuevas-Aburto, J., Janicijevic, D. & Weakley, J. (2019). Assessment of the load-velocity profile in the free-weight prone bench pull exercise through different velocity variables and regression models. *Plos One*, 14(2), e0212085. 10.1371/journal.pone.0212085 PMC6392250 30811432

[ref13] García-Ramos, A. (2023a). The 2-Point Method: Theoretical Basis, Methodological Considerations, Experimental Support, and Its Application Under Field Conditions. International Journal of Sports Physiology and Performance, 18(10), 1092–1100. 10.1123/ijspp.2023-0127 37541677

[ref14] García-Ramos, A. (2023b). Resistance training intensity prescription methods based on lifting velocity monitoring. International Journal of Sports Medicine, 45(4), 257–266. 10.1055/a-2158-3848 37607576

[ref15] González-Badillo, J. J. & Sánchez-Medina, L. (2010). Movement velocity as a measure of loading intensity in resistance training. International Journal of Sports Medicine, 31(5), 347–352. 10.1055/s-0030-1248333 20180176

[ref16] Grgic, J., Scapec, B., Pedisic, Z. & Mikulic, P. (2020). Test-retest reliability of velocity and power in the deadlift and squat exercises assessed by the GymAware PowerTool system. *Frontiers in Physiology*, 11, 561682. 10.3389/fphys.2020.561682 PMC7510176 33013482

[ref17] Haff, G. G. & Dumke, C. L. (2019). *Laboratory Manual for Exercise Physiology* (2nd ed.). Human Kinetics.

[ref18] Haischer, M. H., Carzoli, J. P., Cooke, D. M., Pelland, J. C., Remmert, J. F., & Zourdos, M. C. (2023). Predicting total back squat repetitions from repetition velocity and velocity loss. Journal of Human Kinetics, 87, 167–178. 10.5114/jhk/162021 37229411 PMC10203840

[ref19] Hopkins, W. G., Marshall, S. W., Batterham, A. M. & Hanin, J. (2009). Progressive statistics for studies in sports medicine and exercise science. Medicine & Science in Sports & Exercise, 41(1), 3–13. 10.1249/MSS.0b013e31818cb278 19092709

[ref20] Iversen, V. M., Norum, M., Schoenfeld, B. J. & Fimland, M. S. (2021). No time to lift? Designing time-efficient training programs for strength and hypertrophy: A narrative review. Sports Medicine, 51(10), 2079–2095. 10.1007/s40279-021-01490-1 34125411 PMC8449772

[ref21] McBurnie, A. J., Allen, K. P., Garry, M., Martin, M., Thomas, D., Jones, P. A., Comfort, P. & McMahon, J. J. (2019). The benefits and limitations of predicting one repetition maximum using the load-velocity relationship. Strength and Conditioning Journal, 41(6), 28–40. 10.1519/SSC.0000000000000496

[ref22] Miras-Moreno, S., García-Ramos, A., Fernandes, J. F. T. & Pérez-Castilla, A. (2023a). Lifting more than two loads compromises the magnitude of the load-velocity relationship variables: Evidence in two variants of the prone bench pull exercise. *Applied Sciences*, 13(3), 1944. 10.3390/app13031944

[ref23] Miras-Moreno, S., García-Ramos, A., Jukic, I. & Pérez-Castilla, A. (2023b). Two-point method applied in field conditions: A feasible approach to assess the load-velocity relationship variables during the bench pull exercise. Journal of Strength and Conditioning Research, 37(7), 1367–1374. 10.1519/jsc.0000000000004405 36728020

[ref24] Niewiadomski, W., Laskowska, D., Gąsiorowska, A., Cybulski, G., Strasz, A. & Langfort, J. (2008). Determination and prediction of one repetition maximum (1RM): Safety considerations. Journal of Human Kinetics, 19, 109–120. 10.2478/v10078-008-0008-8

[ref25] Orange, S. T., Metcalfe, J. W., Robinson, A., Applegarth, M. J. & Liefeith, A. (2020). Effects of in-season velocity-versus percentage-based training in academy rugby league players. International Journal of Sports Physiology and Performance, 15(4), 554–561. 10.1123/ijspp.2019-0058 31672928

[ref26] Pérez-Castilla, A., Ramirez-Campillo, R., Fernandes, J. F. T. & García-Ramos, A. (2023). Feasibility of the 2-point method to determine the load−velocity relationship variables during the countermovement jump exercise. Journal of Sport and Health Science, 12(4), 544–552. 10.1016/j.jshs.2021.11.003 34852294 PMC10362485

[ref27] Pestaña-Melero, F. L., Haff, G. G., Rojas, F. J., Pérez-Castilla, A. & García-Ramos, A. (2017). Reliability of the load-velocity relationship obtained through linear and polynomial regression models to predict the one-repetition maximum load. Journal of Applied Biomechanics, 34(3), 184–190. 10.1123/jab.2017-0266 29252060

[ref28] Rebelo, A., Pereira, J. R., Martinho, D. V., & Valente-Dos-Santos, J. (2023). Effects of a velocity-based complex training program in young female artistic roller skating athletes. Journal of Human Kinetics, 86, 217–234. 10.5114/jhk/159654 37181267 PMC10170548

[ref29] Sánchez-Medina, L., Pallarés, J. G., Pérez, C. E., Morán-Navarro, R. & González-Badillo, J. J. (2017). Estimation of relative load from bar velocity in the full back squat exercise. *Sports Medicine International Open*, 1(2), E80–e88. 10.1055/s-0043-102933 30539090 PMC6226068

[ref30] Suchomel, T. J., Nimphius, S. & Stone, M. H. (2016). The importance of muscular strength in athletic performance. Sports Medicine, 46(10), 1419–1449. 10.1007/s40279-016-0486-0 26838985

[ref31] Suchomel, T. J., Cantwell, C. J., Campbell, B. A., Schroeder, Z. S., Marshall, L. K., & Taber, C. B. (2024). Braking and propulsion phase characteristics of traditional and accentuated eccentric loaded back squats. Journal of Human Kinetics, 91, 121–133. 10.5114/jhk/185726 38689588 PMC11057614

[ref32] Thompson, S. W., Rogerson, D., Ruddock, A., Banyard, H. G. & Barnes, A. (2021). Pooled versus individualized load-velocity profiling in the free-weight back squat and power clean. International Journal of Sports Physiology and Performance, 16(6), 825–833. 10.1123/ijspp.2020-0534 33547259

[ref33] Tsoukos, A., & Bogdanis, G. C. (2023). Lower fatigue in the eccentric than the concentric phase of a bench press set executed with maximum velocity to failure against both heavy and light loads. *Journal of Human Kinetics*, 88, 119–129. 10.5114/jhk/168792 PMC10407316 37559769

[ref34] Weakley, J., Wilson, K., Till, K., Banyard, H., Dyson, J., Phibbs, P., Read, D. & Jones, B. (2018). Show me, tell me, encourage me: The effect of different forms of feedback on resistance training performance. Journal of Strength and Conditioning Research, 34(11), 3157–3163. 10.1519/jsc.0000000000002887 33105366

[ref35] Weakley, J., Morrison, M., García-Ramos, A., Johnston, R., James, L. & Cole, M. H. (2021a). The validity and reliability of commercially available resistance training monitoring devices: A systematic review. Sports Medicine, 51(3), 443–502. 10.1007/s40279-020-01382-w 33475985 PMC7900050

[ref36] Weakley, J., Mann, B., Banyard, H., McLaren, H. S., Scott, T. & García Ramos, A. (2021b). Velocity-based training: From theory to application. Strength and Conditioning Journal, 43(2), 31–49. 10.1519/ssc.0000000000000560

[ref37] Weakley, J., Cowley, N., Schoenfeld, B. J., Read, D. B., Timmins, R. G., García-Ramos, A. & McGuckian, T. B. (2023). The effect of feedback on resistance training performance and adaptations: A systematic review and meta-analysis. Sports Medicine, 53(9), 1789–1803. 10.1007/s40279-023-01877-2 37410360 PMC10432365

[ref38] Weir, J. P. (2005). Quantifying test-retest reliability using the intraclass correlation coefficient and the SEM. Journal of Strength and Conditioning Research, 19(1), 231–240. 10.1519/15184.1 15705040

